# Connecting Scientometrics: Dimensions as a Route to Broadening Context for Analyses

**DOI:** 10.3389/frma.2022.835139

**Published:** 2022-04-26

**Authors:** Simon J. Porter, Daniel W. Hook

**Affiliations:** ^1^Digital Science, London, United Kingdom; ^2^Centre for Complexity Science, Imperial College London, London, United Kingdom; ^3^Department of Physics, Washington University in St. Louis, St. Louis, MO, United States

**Keywords:** bibliometrics, scientometrics, Dimensions, Google BigQuery, World Bank data, contextual data, cloud, unique identifier

## Abstract

Modern cloud-based data infrastructures open new vistas for the deployment of scientometric data into the hands of practitioners. These infrastructures lower barriers to entry by making data more available and compute capacity more affordable. In addition, if data are prepared appropriately, with unique identifiers, it is possible to connect many different types of data. Bringing broader world data into the hands of practitioners (policymakers, strategists, and others) who use scientometrics as a tool can extend their capabilities. These ideas are explored through connecting Dimensions and World Bank data on Google BigQuery to study international collaboration between countries of different economic classification.

## 1. Introduction

A foundational focus of bibliometrics and scientometrics has been, both for academics and practitioners, the accumulation of data to support analyses that can be used in resource allocation across the research world and strategic decision making in policy contexts. Understanding the many different aspects of the sociology of the research ecosystem could lead to improved research practices and the ability to target funding more accurately, in turn, driving better outcomes for all. These efforts are significantly impacted and influenced by issues of data coverage (e.g., temporal, subject-based, and geographic extent of the data), as well as in its granularity, accuracy, and standardization. Recent work by the current authors (Hook and Porter, 2021), points out that the ability to access and manipulate these data is a critical component of a healthy research ecosystem and that access to Cloud compute capacity has the potential to transform access to both data and analysis.

Yet, if one is to think deeply about the sociology of research, one should have a clear route to contextualizing the data in bibliometric and bibliographic sources. Research does not exist in a vacuum, it is influenced by both global and local events: Funding for research is dependent on the prosperity of nations, the subject focus of researchers changes in reaction to international circumstances (e.g., COVID-19), and output volumes respond to evaluation environments, metrics, and the stimuli introduced by policy makers, to name just a few “research tropisms.” Thus, we believe that it is important to connect bibliometric data to other external contextual data in order to examine the trends that may drive research through analysis of the correlations between the research world and the wider world.

While correlation does not always imply causation, understanding a broad global picture when examining a complex ecosystem such as the research world, an understanding of global trends remains a critical tool in making decisions. This article explores one potential route to connecting bibliographic and bibliometric data to other cotenxtualizing data (in this case World Bank demographic data). Our approach is not unique—there are both many ways to bring contextualizing data into a study and many different contextualizing datasets that can be of value. Our approach, leverages infrastructure that has been developed by Dimensions and accesses questions that are natural in that setting. We could have looked regional literacy levels, national GDP, or a host of other metrics contained in the World Bank data but chose, for simplicity, to examine the effects of socioeconomic status on international collaboration. In particular, it is worth noting that our analysis says nothing about the quality of research or researchers in any specific location. Rather, the lessons that can be drawn from this study are most likely to highlight issues and correlate with the development of research infrastructure, mobility of researchers, and host of other factors.

### 1.1. The Evolving Data Landscape

The last 10 years have seen significant developments in both the number of sources of bibliometric data that are available (and hence access to bibliometric data to a broad audience) and to the level of accuracy and granularity of those data. Sources such as Microsoft Academic Search, Dimensions, and Google Scholar have joined PubMed, Web of Science and Scopus as significant sources for bibliometric analysis, while source such as Unpaywall have extended these datasets with additional, practical data that is highly relevant for research support systems, policy analysis, and strategic decision making.

Since its launch in 2018, Dimensions has differentiated itself from other tools in the space on several fronts, two of which are relevant in the context of this article, these are: (i) the use of open unique identifiers as a basis for the data held in the data system (and all the consequences of that choice such as the general inclusion of items in the database without additional editorial intervention); and (2) the broadening of the “fundamental dataset” available for scientometric analysis by not only including publication and citation data, but also data on grants, clinical trials, patents, and policy documents in a single interlinked graph (Hook et al., 2018; Herzog et al., 2020). The data structures that naturally emerge when taking this approach to creating Dimensions are well suited to the analysis that we will showcase here.

More recently, the data contained in Dimensions has been made available on the Google BigQuery cloud infrastructure. We believe that infrastructures such as these represent a significant opportunity for the adoption and deployment of scientometric analysis. The use of cloud infrastructure achieves two aims: Firstly, it negates the need to implement large, expensive local processing capabilities, providing on-demand access to computational power at a fraction of the cost of local implementations; and, secondly, it facilitates new modes of data sharing, allowing the mixing of public and private datasets in a secure environment. Both of these facets of cloud compute serve not only to democratize access to analysis (Hook and Porter, 2021), but also facilitate an increasingly iterative and real-time relationship with analysis (Hook et al., 2021).

Research is an intrinsically and increasingly collaborative activity with an increasingly global set of norms and infrastructures (Tijssen et al., 2011; Waltman et al., 2011). Adams (2013) demonstrates that research is becoming a more international endeavor. While the center of this analysis is concerned with the UK, it is clearly more generally applicable and justifies Adams' claim that we are entering a fourth age of research where the normal mode of research is international rather than individual (first age), departmental (second age), or national (third age).

**Figure 1:**
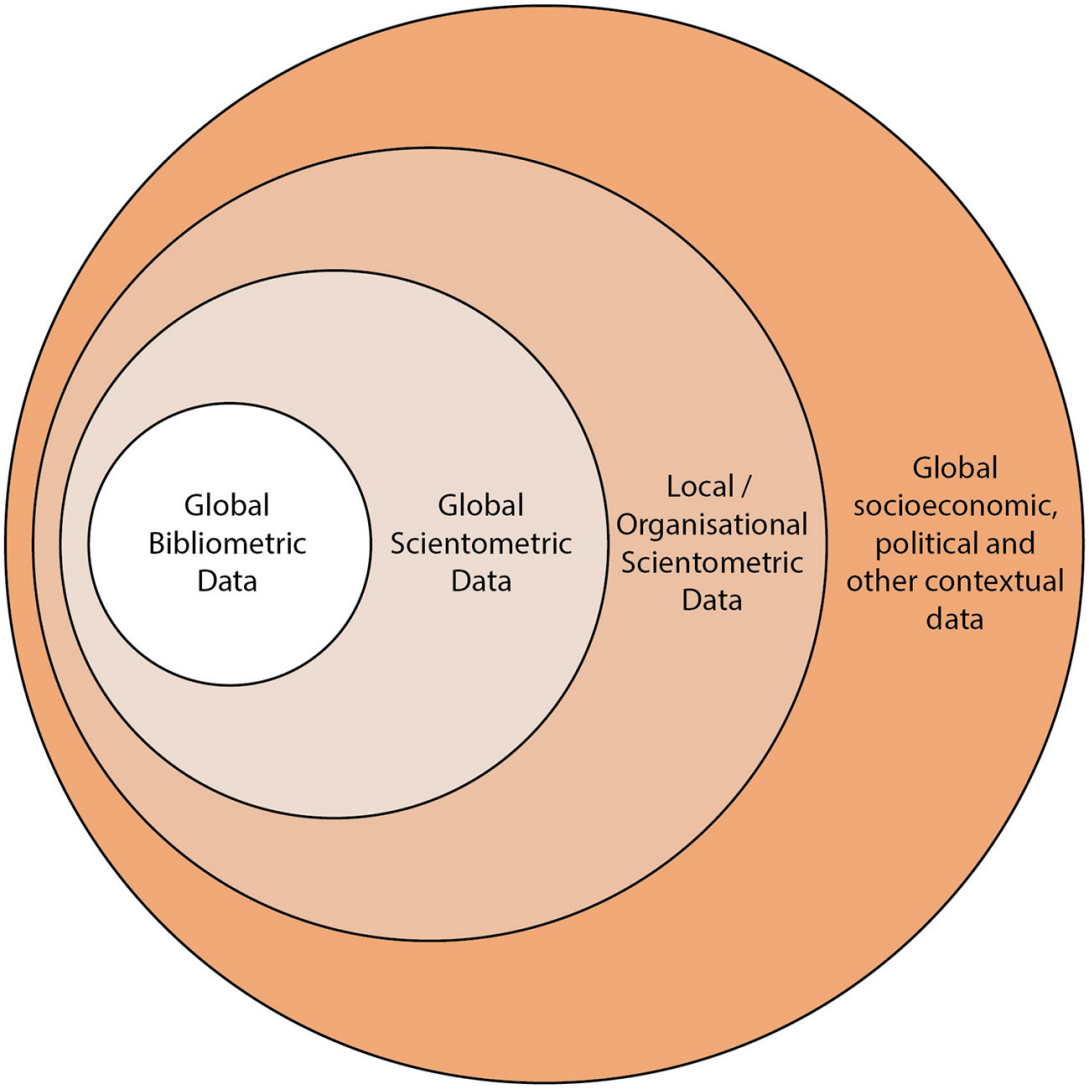
Four modes of data use in scientometrics. The most general data, often used for the purposes of resource planning and policy is Global Bibliometric Data—it consists of the bibliographic data such the co-authorship graph, the locational information regarding journal reference and the geographic information. It is the least detailed data and often has the least coverage to PID infrastructure of the datasets shown in this diagram. Global Scientometric Data extends the Global Bibliometric Data with the citation graph and may include altmetric data, although this could also be argued to sit in the outer circle depending on whether it is viewed as attention or context. Local/Organizational Scientometric Data is the data contained in local CRIS systems or national repositories. This data is often enhanced by curation by institutional, funder-based or academicians themselves. Any of these three inner circles may be enhanced by subject classification or annotation approaches to add new facets to the data. The outer circle contains global socioeconomic, political, and other contextual data, which is not typically viewed as being part of the scientometric world. In these data we include items that may connect scientometric data through place, time, or other locational information the the broad world.

In analogy with Adams' “Fourth age of Research,” we argue that scientometrics has four different modes of data use (see Figure 1). The simplest mode is use of global bibliographic data to do high-level analysis such as the construction of benchmarks or the assessment of national, institutional, or subject-based research volumes. We don't include the citation graph in this definition of the first mode—it is concerned with volume-based measures and co-authorship-style analyses. With the introduction of databases such as the Web of Science in the 1950s, this first mode was the early, and most accessible, way to being to study the sociology of research. The next simplest mode is use of broader data about the research ecosystem. It adds the citation graph to the first mode. Use cases are similar to the first mode—benchmarking or general contextualization of research inside a scholarly context. The addition of the citation graph adds significant capability and insight as the lineage of research can be established.

The third mode is the addition of organizational or local data in analyses: While the first two modes concern global datasets, this mode adds a local reference dataset that is either being analyzed in isolation or that is being contextualized using the scholarly datasets and approaches from modes 1 and 2. Third-mode data are often sensitive in nature and include some of the data held in institutional CRIS or RIMS systems and in funder systems: These data may including funding success rates, data that is personal to the researcher such as ethnicity or gender, or details of industrial funding. These data start to branch outside the standard sphere of bibliometric analysis and bring important context to the analysis that can take place inside an academic institution, but are naturally difficult to share outside an institutional setting. Some of these data are also part of national exercises but suffer from the same challenges for sharing.

The fourth mode concerns broader contextualization and connection beyond a purely academic or scholarly considerations. These data are “safer” than mode 3 data as they contain fewer personal items. Examples of data and modes in this fourth category include altmetric data (public engagement with research) (Sugimoto et al., 2017), awarded-grant data, patent data and clinical trials data. These data have the capacity to reflect the environment in which research is taking place and should facilitate the connection to the wider non-research-centric world. As such, we consider socioeconomic data to also sit in this class of data. To understand the GDP of a country, local literacy rates, public spending on education, trends of researchers per capital and a host of other metrics, is to understand the level of development of the research infrastructure in the place where research is taking place.

We assert that policy makers and good strategists naturally attempt to access a mode of scientometric analysis that seeks to bring greater context to their analyses (see Figure 1 outer circle). There are many examples of researchers who take this broader view—often inspired by or originating from economics as a discipline (Lane, 2009, 2010; Lane and Bertuzzi, 2011). However, getting data in a consistent format and at a level of quality that admits such analyses is challenging and is often the focus of significant research projects in and of itself.

The approach that we demonstrate in this article does not negate the challenges of sourcing, curating, and manicuring data for quality. Indeed, recent studies (Guerrero-Bote et al., 2021; Porter, 2022) illustrate the challenges of ensuring data quality in address disambiguation even through there is clearly an ongoing effort to improve these data in all parts of the scholarly information ecosystem. However, it does attempt to showcase a new set of technologies and techniques for re-using data in a broad range of applications and connecting datasets together. We regard unique identifiers as an enabling infrastructure that allows multiple datasets from different origins to be bought together quickly and easily.

In the example use case that we present here, we connect World Bank data as a global economic dataset to Dimensions as a scientometric dataset on a country level using ISO country coding as our gateway. Our use case is not novel in scope as many other authors have carried out similar studies, see for example (Gazni et al., 2012; Chetwood et al., 2015), but the methodology that we introduce is. We believe that it demonstrates the potential to perform sophisticated analyses with great speed and opens up this possibility to many at low cost through the use of Cloud-based infrastructures, and as such represents an opportunity to improve the application of bibliometric and scientometric analysis in a much broader range of policy areas.

Our motivation for choosing the present example is that collaboration is a core from which many different interesting research questions can be asked. Beyond basic quantification of volumes of collaboration or geographic and institutional loci of collaboration, classification of modes of collaboration is intrinsically interesting. The Dimensions dataset already contains the data to quantify the attention associated with collaboration (both public attention through Altmetric.com data and scholarly attention from citations), the financial support behind collaboration (grant data), the impact of collaboration (patents, clinical trials, policy documents), and the fields of research being explored in the research. By connecting to World Bank data we can go beyond these purely scientometric considerations and leverage any of the 1,442 indicators that the World Bank makes available on the Google BigQuery data marketplace.

This article is arranged as follows: In Section 2, we give an overview of the computational infrastructure that supports this article, the data infrastructure used for the examples shown and we share basic code listings that highlight the brevity needed to perform these analyses. In Section 3, we present some initial results gained using the techniques that are the focus of this article. In Section 4, we provide a few thoughts on both the nature of the results and the ease of their production.

## 2. Methods

### 2.1. Infrastructure

Use of cloud infrastructure is at the core of this article. While we have chosen to use:

- Compute/Data Infrastructure: BigQuery on Google Cloud
- Bibliometric/Scientometric data: Dimensions
- Programming Language: Python

All these choices could be exchanged for equivalents. The compute and data infrastructure provided by Google has analogues from Amazon, Snowflake, Microsoft, Tencent, and others. Bibliometric and Scientometric data can be sourced from an increasing array of providers. We chose to use the Google Colaboratory as our development environment and used Python by default. However, similar analyses can be carried out using other languages and even business intelligence tools such as Tableau.

### 2.2. Open Data Standards

At the core of the methodology of the work reported in this article are open data standards. The Dimensions dataset is built on open unique identifiers wherever possible. When Dimensions was first built, there was no publicly available system of unique identifiers associated with organizations that perform and publish research. As a result, the Digital Science team created GRID^1^. Dimensions on BigQuery includes mappings from each of the research object data types to GRID where an institutional affiliation can be resolved.

At the time of publication the GRID dataset includes more than 120,000 different research organizations with, among other pieces of metadata, geographical information about the principle campus of each organization. GRID attempts to be a gateway to many different standards, which might be helpful when performing analyses, for example: NUTS coding information, geographical longitude, and latitude location of the principle campus and mapping to ISO 3166-1 alpha-2 country codes.

More recently Digital Science announced that GRID would not be making any further public releases as the Research Organization Registry (ROR) has built sufficient support in the community that it has become the principle organizational identifier. Both GRID and ROR maintain a mappings between the two identifier systems to ensure maximal interoperability. For simplicity of exposition in the examples shown in this article, GRID is used, however, RORs could be used at the preference of the researcher.

For the study represented in this article, the World Bank country income classifications that were updated in July 2021 were used. Each year these classifications are updated. There are four incoming groups: Low, lower-middle, upper-middle, and high-income countries. These classifications relate to the annual Gross National Income (GNI) in USD calculated using the Atlas method exchange rates (World Bank, 2021).

At the time of performing this analysis, the classification levels are defined in Table 1.

**Table 1:** Definitions of World Bank income level classifications 2021–2022 (World Bank, 2021).

**Group**	**GNI per capita (USD)**
Low income	<1,046
Lower-middle income	1,046–4,095
Upper-middle income	4,096–12,695
High income	>12,695

For the purposes of this article, even though we look at data slices over a longer period of time (typically a 10-year period) we have only used the income classifications from 2021. Our methodology could be improved if we were to track the changes in world bank country classification as publication output changes take place, however, these changes would not significantly effect the outcome of the analysis presented here. Since our focus is on demonstrating the methodology we have decided that it would be more confusing to present this extension to the analysis than it would be to demonstrate the method using the simpler approach.

### 2.3. Data Structure

**Figure 2:**
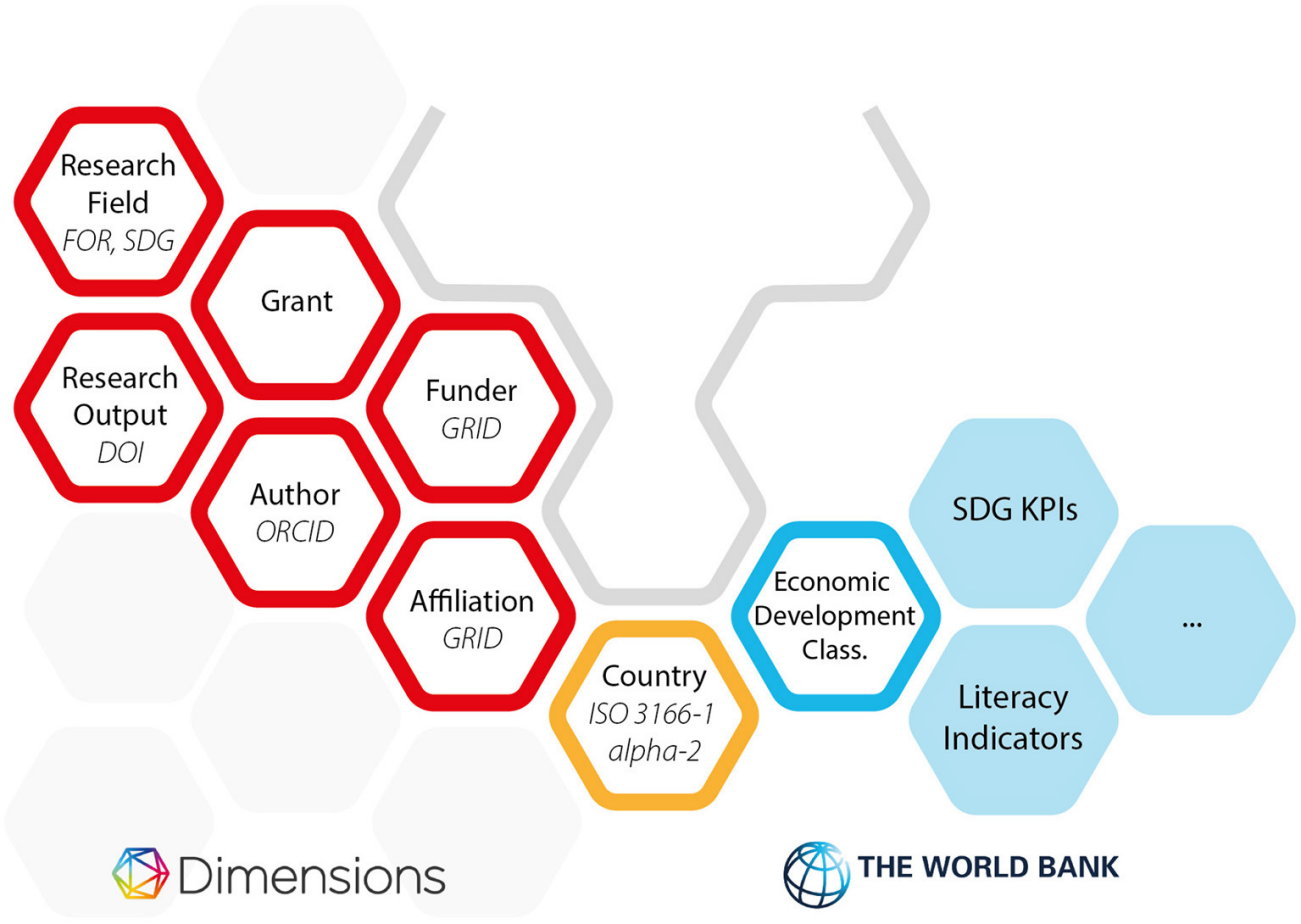
High-level entity diagram showing the objects in the Google Big Query environment that support Listing 1. Hexagons outlined in red are those contained in the Dimensions dataset. Hexagons outlined in blue are the data from the World Bank Dataset that are used in this article. Hexagons in solid blue are other hexagons in the World Bank Dataset that haven't been used in this article but which could be of significant interest in further studies. The hexagon outlined in gold is the data that appears in both dataset allowing the link and contextualization that we explore in this article. For those wishing to explore this further, Dimensions has released a free COVID-19 dataset on the Google Cloud Marketplace, which has identical structure to the main database.

Figure 2 shows a schematic representation of two sets of tables in the Google BigQuery environment. Each hexagon is a notional data table in the BigQuery environment apart from the yellow “country” hexagon, which has been picked out as the key linking piece of data that connects the Dimensions data (red hexagons) to the World Bank data (blue hexagons). The solid blue hexagons are examples of other data in the World Bank dataset that are not used in this article. The Dimensions data shown here is in the private space of Digital Science (access available *via* subscription)^2^. The World Bank data is publicly available in the BigQuery Marketplace.

In each case, where there is either a unique identifier or public dataset that is a key for one of the Dimensions tables, this has been picked out in italic letters in the hexagon. For example, researchers in Dimensions are linked to ORCIDs, Research outputs of various types are linked to DOIs from Crossref or DataCite. Full details of the Dimensions data structure and approach can be found in Hook et al. (2018).

### 2.4. Query Approach

The analyses shown in the results section are all based on the same core query, which is shown in Listing 1. Note that this code is highly economical—the code produces a table listing the number of fully normalized co-authorships between countries by income classification based on publications from the period 2010 to 2020 and also prepares the total number of co-authored international publications in a final column, to allow the calculation of percentages. The total code (minus comments) is under 30 lines and executes almost immediately on the Google BigQuery infrastructure.

**Listing 1:**
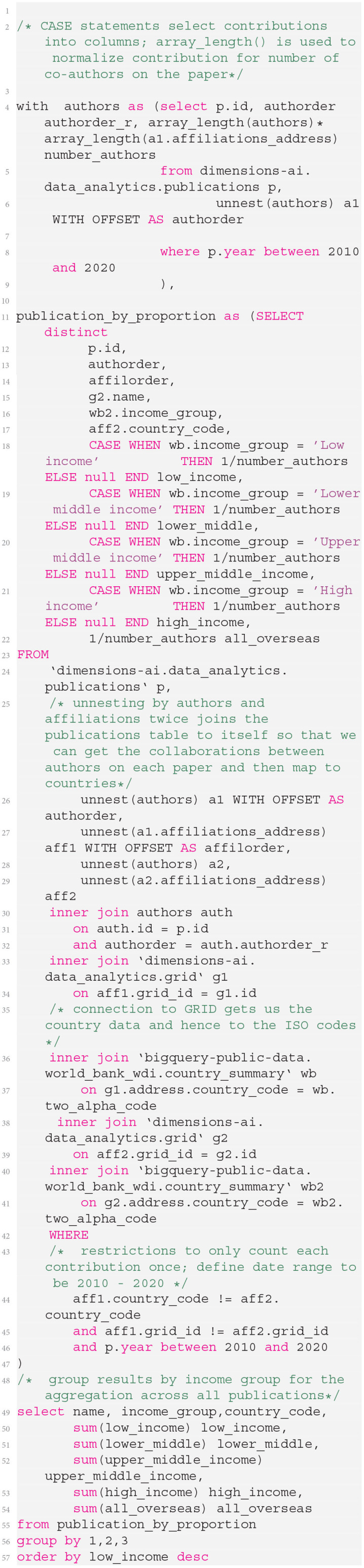
Listing to produce an author-contribution-weighted summary of collaborations between countries and connect this to World Bank data using the Google Big Query environment.

## 3. Results

In this section, we briefly show some of the results that can be rapidly obtained from the approach that we have described above. In these examples, we use different facets of the Dimensions data to show some of the capability that is available while only using one of the many tables available in the World Bank dataset. Thus, these examples merely hint at the opportunity for detailed, real-time analysis.

Each set of results shown below is fruit of taking the coding in Listing 1 and joining on one of the Dimensions tables shown in red in Figure 2.

**Figure 3:**
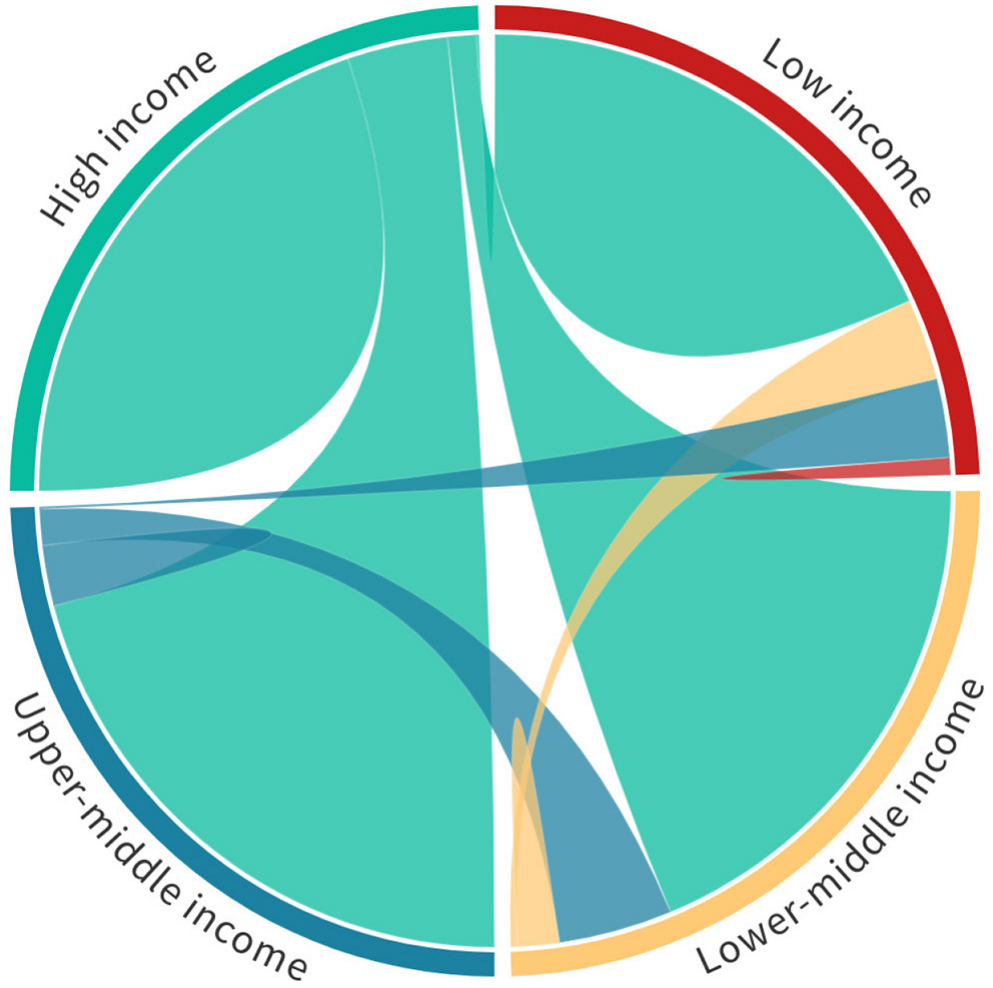
Global collaboration between high-income, upper-middle-income, lower-middle-income, and low-income countries on publications between 2010 and 2020. Each quadrant in the chord diagram corresponds to 100% of the research output of countries in each of the brackets. Thus, low-income levels of output have not been normalized in proportion to that of lower-middle, upper-middle, and high-income countries. In this view we see the proportion of collaboration between different economic bands (defined in Table 2). A chord diagram visualisation is helpful to understand the interplay between bi-lateral relationships, but it is important to note that the representation is not entirely faithful as it cannot include multi-lateral relationships directly – a higher-dimensions representation would be required to encapsulate these relationships. However, the contributing bi-lateral component of a multi-lateral collaboration do contribute to each arc.

To begin we have calculated the relative levels of collaboration between countries of different World Bank economic classification level based on papers written between 2010 and 2020 (see Figure 3 and Table 2). In this figure (and the corresponding table) all domestic papers have been removed. This means that interactions of, for example, low-income to low-income countries are interactions between different low-income countries.

**TABLE 2:** Proportion of collaborative output between 2010 and 2020 for global outputs by World Bank country classification level.

**% of output**	**Low**	**Lower-middle**	**Upper-middle**	**High**
Low	2.91	13.43	14.16	69.49
Lower-middle	0.91	6.70	18.46	73.92
Upper-middle	0.38	5.31	16.58	77.70
High	0.28	6.24	9.64	83.74

*Rows add to 100%*.

Even from this simple analysis it is clear that high-income countries dominate international collaborative relationships with all other economic brackets. Perhaps more interesting is that there appears to be a somewhat unexpected symmetry in that each of the low, lower-middle, upper-middle, and high income countries all have approximately the proportion between high-income interactions and interactions collectively involving low, lower-middle and upper-middle categories. Table 2 shows the detailed proportions shown in Figure 3.

Perhaps unsurprisingly, the lower the income level of the country, the less engaged they are able to be in the global research community, but it is interesting that low-income countries still make up almost 2.5% of their collaborative output with other low-income countries. Another interesting facet shows that upper-middle-income countries are those most engaged with High income countries—presumably because they are beginning to have their own resources as their research economies develop, hence, the engagement is less unidirectional.

**Figure 4:**
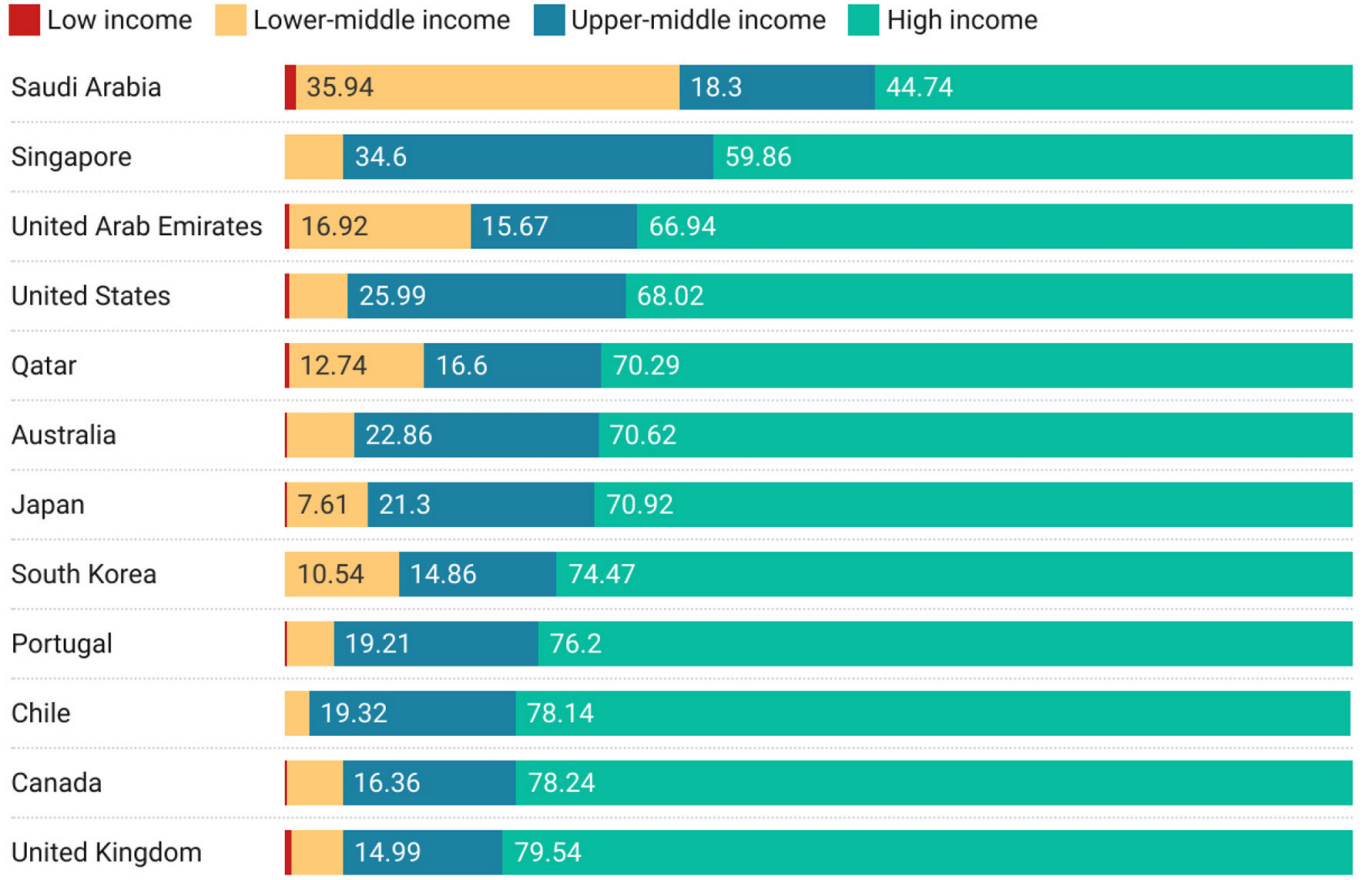
Top 12 high-income countries with the highest non-high-income country collaborations, ordered by amount cumulative proportion of non-high-income collaboration.

Having setup this basic framework, many different directions of enquiry are available to us. For example, we might wish to understand which high-income countries are the most collaborative with countries of lower income levels. Figure 4 shows the top 12 high-income countries by level of non-high-income country collaboration. This figure is ordered by the highest level of non-high-income collaboration and hence does not emphasize low-income collaborations. No specific geographic focus emerges from the high-level data featured in Figure 4. There may be specific geographic patterns that suggest themselves on further analysis of the data—geographic proximity to countries in lower economic brackets may be one theory. In the case of the UK and the US, there may be historical or linguistic forces at play—a more detailed study would be needed. We merely point out that this analysis is a gateway to ask such questions.

**Figure 5:**
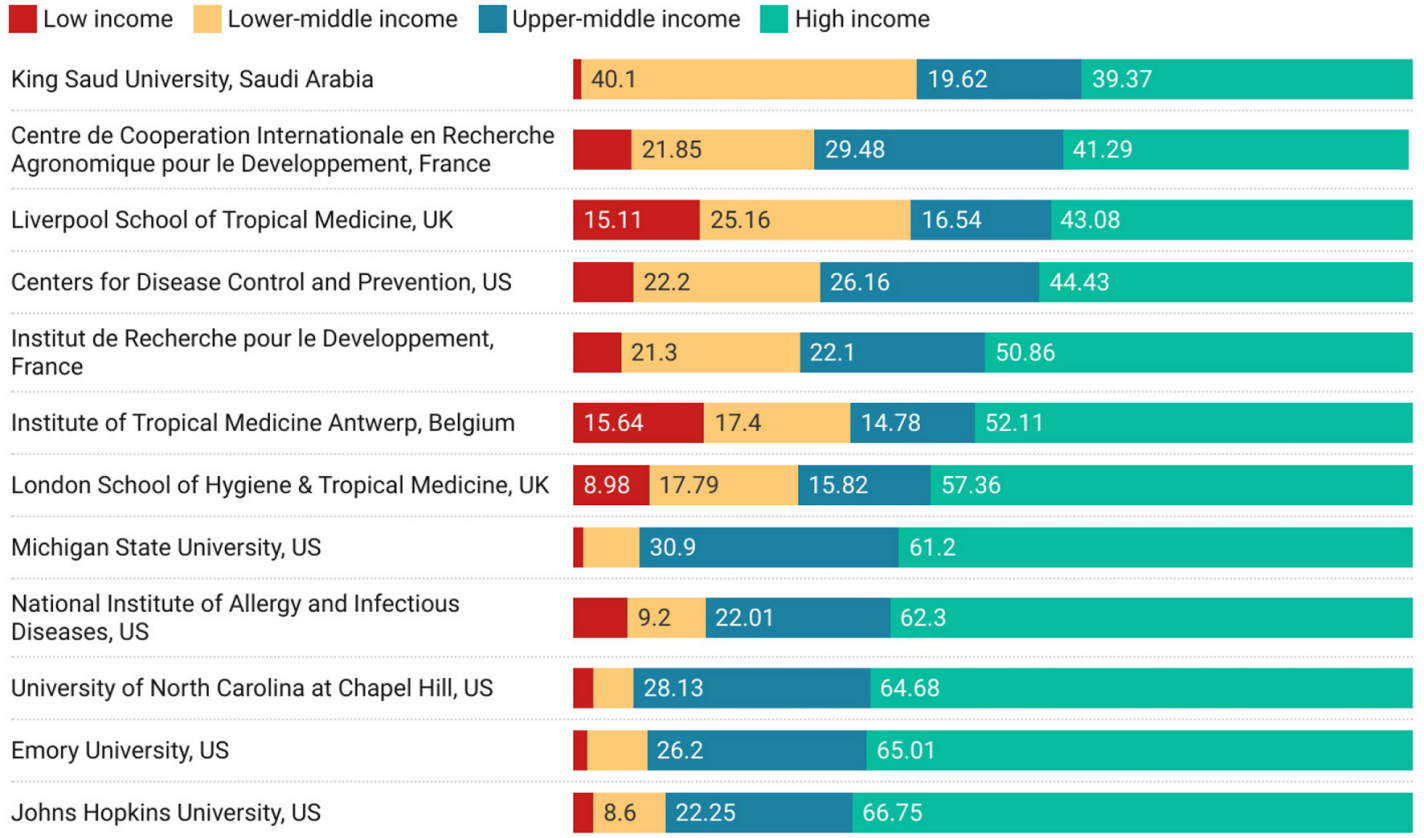
Top 12 High-income-country research institutions with highest non-high-income collaborations.

A similar analysis can be performed at an institutional level, which is shown in Figure 5. In this figure, almost all the previous countries disappear from the analysis showing that the ability to quickly change between different aggregating objects (countries to institutions) is an important feature of iterative exploratory analysis. However, this figure suggests an alternative line of enquiry—the names of institutions are suggestive of subject biases that might lead to preferred relationships with developing economies—particularly around medicine and tropical diseases.

**Figure 6:**
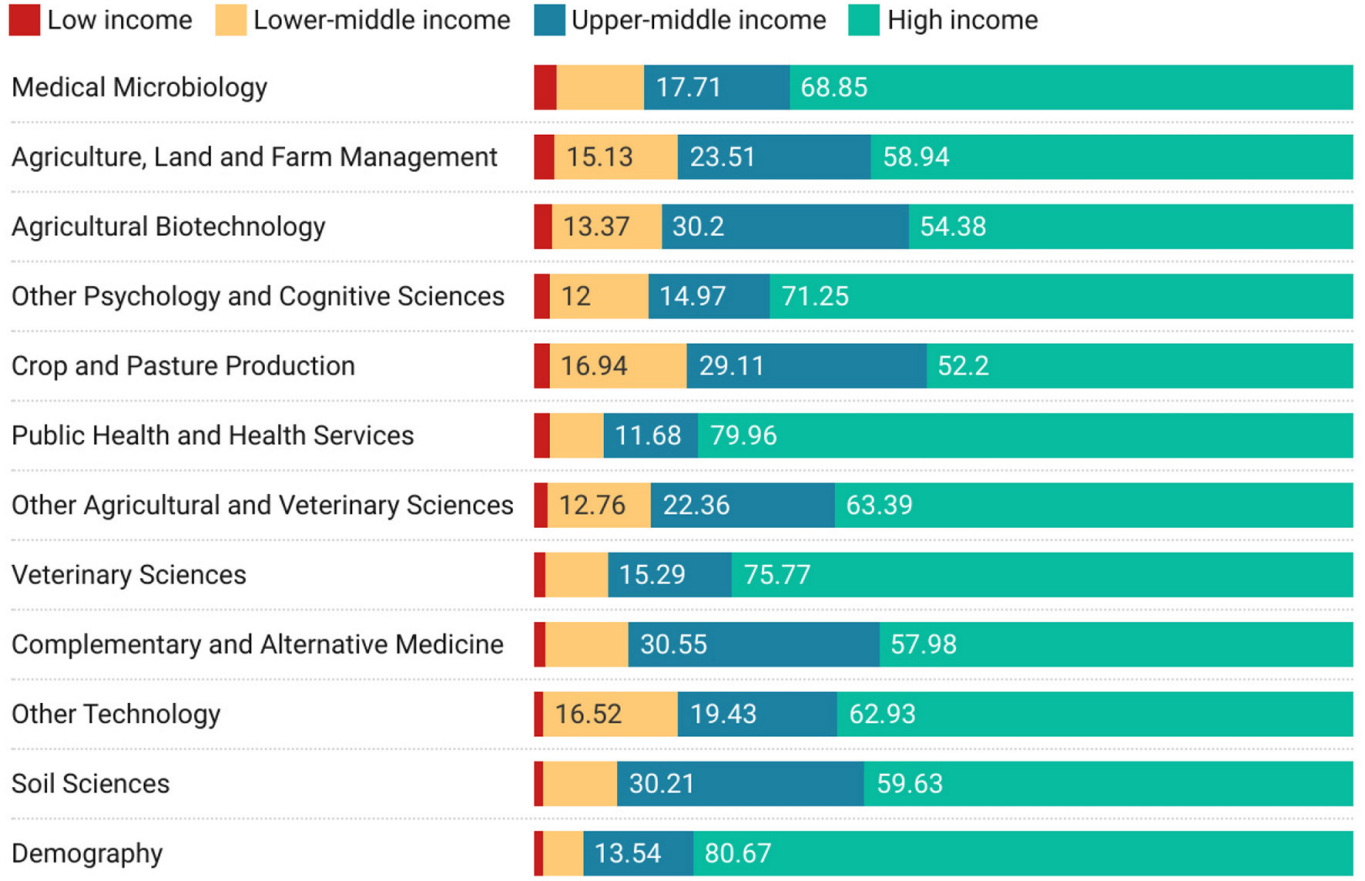
Top 12 high-to-Low-income-country collaborations by field of research ordered by proportion of low-income country collaborations.

Figure 6 begins to explore the subject areas that high-income countries collaborate on by economic class using the ANZSRC Field of Research Code classification. Ordered by participation with Low income countries, this figure suggests that medical, agricultural, and sociological collaborations may be the mainstay of collaborations between high income and low income participants.

**Figure 7:**
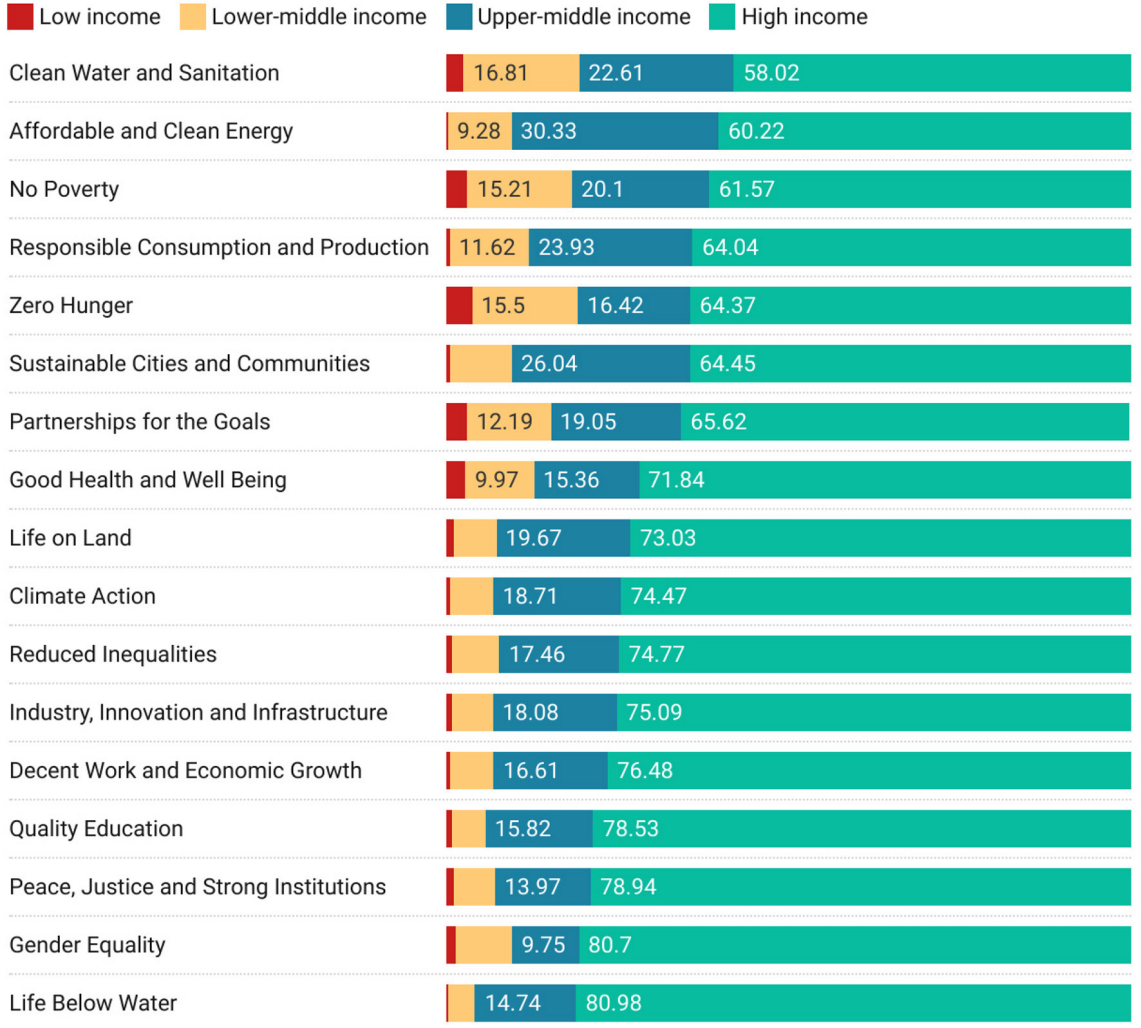
Sustainable development goal collaborations from the perspective of low-income countries, ordered by cumulative non-high-income proportion of research.

To add a slightly different perspective, we examine which economic classifications of partner are working with low-income countries on different United Nations Sustainable development goals (SDG). Figure 7 shows how much collaboration is taking place between low-income countries and other economic levels by SDG. Hence, we can see that the largest collaboration with higher income countries (as well as with High-income countries specifically) is on the Clean Water and Sanitation SDG.

**Figure 8:**
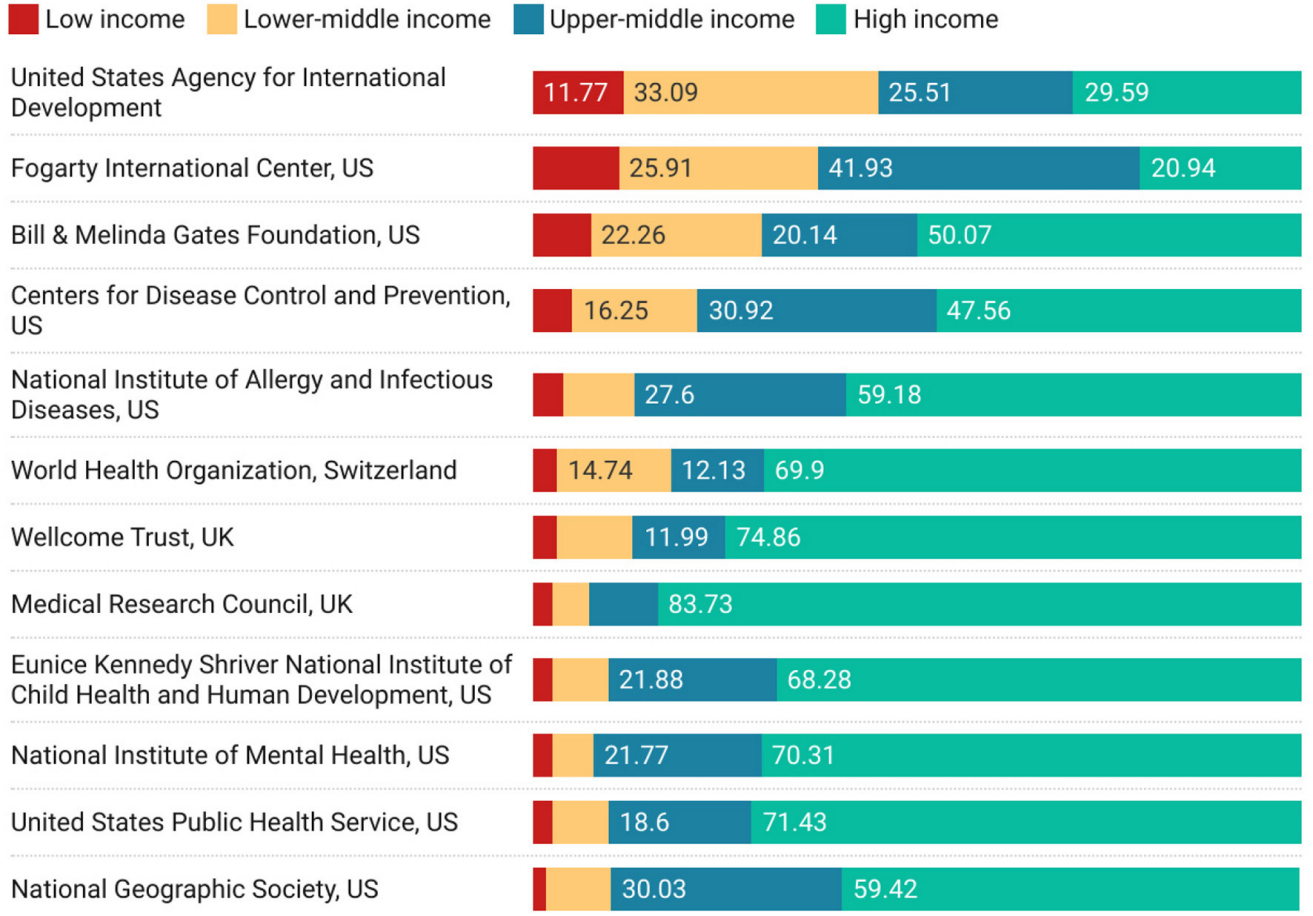
Funding acknowledged in papers co-authored between high-income countries and lower income groups, ordered by proportion of low-income research.

Finally, in Figure 8 we report on funding, from the perspective of high-income countries. The figure shows the worlds funders who have the highest proportion of the international outputs in which they are acknowledged associated with collaborators in countries of lower economic status (ordered by largest proportion of low income collaborations).

## 4. Discussion

### 4.1. Discussion of Results

While the main point of this article is to show the capability of the infrastructure that underlies the results shown here, we start this discussion section with a brief set of observations regarding the analysis in Section 3. In most cultures research is viewed as a positive force that enables economic prosperity and which helps address challenges both practical and intellectual in many different areas from industry to medicine and sociology to art. At the core of research is collaboration with others. We have presented an analysis in which we have focused entirely on proportion and not on volume. This could be considered a weakness of the approach. Yet this lens does offer some interesting insights. For example, it may be surprising that low-income countries do not to work overly with other low-income countries: If higher-income countries are not willing to work with them then perhaps that would be keen to work between each other. This comment is, however, a naïve one, since it is likely not merely a choice not to work together (even on items of common interest such as SDGs), but a more fundamental systemic issue. We suggest that may be a variety of drivers behind this lack of engagement including: geographic separation; developing rather than developed research infrastructure to support international collaboration such as dedicated travel bursaries, international fellowship schemes, and technological infrastructures to support online collaboration; lack of links at a geopolitical level that either hinder or simply do not promote the establishment of links and relationships through researcher mobility that can support ongoing research relationships (Chikanda et al., 2016; Robinson-Garcia et al., 2019; Wagner, 2019; Robinson-Garcia and Ràfols, 2020; OECD, 2021). However, from our analysis, Figure 7 makes it clear that, even when viewed from the perspective of low-income countries themselves, engagement with other low-income countries is relatively lower—and on topics that are important and potentially highly meaningful to low-income countries. However, rather than being a question of bias or exclusionary practices the persistence of this results across many lenses suggests, as mentioned above, deeper systemic issues for low income countries around the fundamental capacity to engage in an international mode, be it due to the development sufficient research capacity, formal research infrastructures around engagement of exclusionary practices in more developed nations (Koehn and Obamba, 2014a,b).

Two figures show a marginally different spectrum of engagement—Figure 5, for institutions and Figure 8 around funding. In Figure 5, we see that while it may be difficult for low income countries to engage at a macro level there can be significant collaboration on specific topics with specific institutions. It is unsurprising that a different collaborative behavior emerges around disease control, and medicine, as can be seen from the names of the institutions listed in the figure (or the current reputation for medical prowess of the institutions in the list). In Figure 8, we see that funders with specific missions are also successful at directing their funding toward supporting collaboration between high income and low income countries.

### 4.2. Reflections on Infrastructure

In this short article, we have attempted to show the potential for the use of modern technical infrastructures in bibliometric and scientometric analysis and we have suggested a framework to think about the different modes of data usage. Much that we have demonstrated may be of interest to the research community as a means to translate the technologies and approaches that they develop to be used broadly by practitioners. The enabling infrastructures (such as unique identifiers) that are described here are well known in both the research context and the practitioner context, yet the allied enabling platforms such as Google BigQuery are, as yet, less well used. The exciting opportunity for researchers is the datasets that they create as part of their research projects may gain additional use and generate additional value to the broader academic community if they are constructed with good use of open data standard and can be shared on infrastructures such as those described here.

The beneficiaries of the technologies described here are manifold. Research policy makers, strategists, and decision makers need to be able to bring their analyses in contact with broader global considerations such as those from financial, economic, and sociological sectors. The technology approach makes that more tractable for those who lack significant resources (to build their own compute and data handling infrastructure).

The notion that research should exist in a broader context and that in order to do that it needs access to well-structured “computable data” is not a new one (Wolfram, 2010). The Mathematica and Wolfram Language system has created an ecosystem where researchers can call upon well-structured contextualizing statistical content has been in place for some time, having been introduced in Mathematica in 2007 (Wolfram Research, 2021). The team has gone on to include domain-specific data in a dizzying array of different fields. However, this ecosystem does not allow the easy addition of new data sources and requires users to be comfortable with the Wolfram Language. While the data in Google BigQuery and other similar environments may be less well structured than the data in the Wolfram environment, it is more easy to contribute to leverage a mix of private and public datasets through open identifier schemes and, due to the nature of the underlying technology approach, these new platforms are all but language agnostic.

Finally, we reflect that we have not seen extensive analysis that focus on the issues highlighted by the analysis that we performed as an example in this article in the general bibliometric or scientometric literature and believe that a significant detailed study is required that moves beyond collaboration volume or attention to the modes of collaboration highlighted in our brief analysis here. We believe that there is a rich seam of data that can no easily be explored through the techniques that we have shared in this article.

While we have focused in this article on one single facet of World Bank data, there are many other tables within the World Bank dataset that can be explored. There are also many other datasets that have been prepared to be used in the ways described in this article. Google's cloud marketplace already includes public datasets from the Centers for Disease Control, the Broad Institute, the United States Census Bureau, and many others.

We believe that analyses such as these have the capacity to empower practitioners to highlight and address issues of significant importance beyond purely scholarly concerns. Connecting scientometrics to broader datasets using the types of methods shown here explores just a fraction of the potential that could be accessed by policy makers at all levels a set of invaluable, accessible tools to make better cases for support, and to make better decisions.

## Data Availability Statement

The datasets presented in this study can be found in online repositories. The names of the repository/repositories and accession number(s) can be found at: https://doi.org/10.6084/m9.figshare.17197262.v1.

## Author Contributions

Ideas for this article were generated and refined by SP and DH. Data analysis for this article was performed by SP. Interpretation of analysis was performed by all co-authors. The article was drafted by DH. Both authors collaborated on editing the article and responding to referee comments.

## Conflict of Interest

SP and DH were employed by Digital Science, the creator and provider of Dimensions.

## Publisher's Note

All claims expressed in this article are solely those of the authors and do not necessarily represent those of their affiliated organizations, or those of the publisher, the editors and the reviewers. Any product that may be evaluated in this article, or claim that may be made by its manufacturer, is not guaranteed or endorsed by the publisher.

## Footnotes

^1^ http://grid.ac

^2^The Dimensions COVID-19 dataset has the same structure as the usual Dimensions data and is freely available on the same infrastructure.
